# The ventral hippocampus is activated in olfactory but not auditory threat memory

**DOI:** 10.3389/fncir.2024.1371130

**Published:** 2024-02-27

**Authors:** Tayebeh Sepahvand, Samantha J. Carew, Qi Yuan

**Affiliations:** Faculty of Medicine, Memorial University of Newfoundland, St. John’s, NF, Canada

**Keywords:** learning and memory, hippocampus, threat conditioning, olfactory, context, auditory

## Abstract

Hippocampal networks required for associative memory formation are involved in cue- and context-dependent threat conditioning. The hippocampus is functionally heterogeneous at its dorsal and ventral poles, and recent investigations have focused on the specific roles required from each sub-region for associative conditioning. Cumulative evidence suggests that contextual and emotional information is processed by the dorsal and ventral hippocampus, respectively. However, it is not well understood how these two divisions engage in threat conditioning with cues of different sensory modalities. Here, we compare the involvement of the dorsal and ventral hippocampus in two types of threat conditioning: olfactory and auditory. Our results suggest that the dorsal hippocampus encodes contextual information and is activated upon recall of an olfactory threat memory only if contextual cues are relevant to the threat. Overnight habituation to the context eliminates dorsal hippocampal activation, implying that this area does not directly support cue-dependent threat conditioning. The ventral hippocampus is activated upon recall of olfactory, but not auditory, threat memory regardless of habituation duration. Concurrent activation of the piriform cortex is consistent with its direct connection with the ventral hippocampus. Together, our study suggests a unique role of the ventral hippocampus in olfactory threat conditioning.

## 1 Introduction

The hippocampus has long been recognized for its critical role in context-dependent threat conditioning and extinction (Maren et al., 1997, 2013; Maren and Holt, 2004; Lacagnina et al., 2019). More recently, functional differences along its dorsal-ventral axis have been appreciated (for review see Fanselow and Dong, 2010). The dorsal hippocampus (DH) is crucial for encoding contextual information in context- and cue-dependent associative memory (Phillips and LeDoux, 1992; Barrientos et al., 2002), while the ventral hippocampus (VH) conveys information about stress, affect, and emotion (Bast et al., 2001; Kjelstrup et al., 2002; Maren and Holt, 2004; Kim and Cho, 2020) via its direct connection with the basolateral amygdala (BLA; McDonald and Mott, 2017). The VH is interconnected with sensory circuitry (Cenquizca and Swanson, 2007; Tao et al., 2021) and has extensive reciprocal connections with the olfactory bulb (Padmanabhan et al., 2018) and piriform cortex (PC; Cenquizca and Swanson, 2007; for review see Fanselow and Dong, 2010), while VH projections to the auditory cortex are sparse (Cenquizca and Swanson, 2007). The unique contribution of the VH to threat conditioning using cues of different sensory modalities is relatively unexplored. Ventral, but not dorsal, hippocampal lesions impair the temporal discrimination of olfactory but not visual or spatial cues (Hunsaker et al., 2008), indicating that the VH may preferentially process cues based on their sensory features. The extensive connectivity of the VH with both olfactory (Padmanabhan et al., 2018; Tao et al., 2021) and threat (Deji et al., 2022) circuitry may support its role in olfactory threat learning. Here, we investigate the activation of the DH and the VH in parallel with the amygdala and relevant sensory cortices in two types of cued threat conditioning, olfactory and auditory, to explore the unique contributions of the DH and the VH in threat learning.

## 2 Materials and methods

### 2.1 Animals

Sprague Dawley rats (3–6 months old, weight 400–900 g, in good health) of both sexes (n = 68 total) were assigned randomly to groups. Sex assignment was balanced in each group. Rats were singly housed in polycarbonate cages on a 12-h light/dark cycle with *ad libitum* access to food and water. All procedures were approved by the Memorial University Institutional Animal Care Committee and carried out in compliance with the guidelines of the Canadian Council on Animal Care.

### 2.2 Apparatus

Context A consisted of a custom-made olfactometer for air and odorant delivery attached to a plexiglass chamber that was positioned atop an electrified grid, connected to a shock generator/scrambler (Muromachi Kikai Model SGS-003DX). Polyvinyl carbonate bottles were used for each odor and connected to the olfactometer by C-flex tubing. Evacuation tubing with a fan was attached to the top lid to promote odor removal. Background white noise of 60 dB was played in the behavioral rooms during experiments. For auditory conditioning experiments, a pure tone (2 kHz, 80 dB) was played using computer speakers connected to a laptop which were placed on opposite walls outside of the conditioning chamber so the animals could not see them.

Context B was a plexiglass chamber covered in a checkerboard pattern in a separate behavioral room from Context A. Odor was delivered by soaking filter paper in odor and placing small pieces inside of fenestrated 15 mL conical tubes adhered to the corners.

In all cases the context was thoroughly cleaned with 70% ethanol between exposures and residual odor was extruded for at least 15 min before the next exposure.

### 2.3 Odorants

Terpinene was diluted with mineral oil (6.63%) so that delivery would emit a vapor-phase partial pressure of 1 Pascal (Devore et al., 2013).

### 2.4 Habituation

For standard habituation (Hsd), rats were placed in Context A for one 30 min session each on two consecutive days immediately prior to training. To avoid any negative association to the experimenter, rats were handled for 5 min each before entrance to and upon exit of Context A. For overnight habituation (Hon), rats were placed in Context A with hydrogel and food pellets and remained inside overnight followed by training the next morning in Context A.

### 2.5 First order odor conditioning

Rats were trained individually with four separate exposures to either odor (O only) or odor and shock (O/S pair) at 5, 15, 20, and 30 min during a 30 min training session in Context A. Odorant (terpinene) was delivered for 1 min at each time point and co-terminated with a shock (0.5 mA for 1 s). “O only” animals were exposed to the odor in the same manner without being shocked. Animals were returned to their home cages immediately following the final odor exposure.

The day after training (recall day) all rats were individually exposed to terpinene (CS1) in Context A for 5 min while recording freezing behavior.

### 2.6 First order context/second order odor conditioning

On day 1, control (context only during 2nd order conditioning; C only) and experimental (odor and context paired; O/C pair) rats were first order conditioned individually in Context A with four separate shocks (0.5 mA for 1 s) at 5, 15, 20, and 30 min during a 30 min training, giving Context A the value of conditioned stimulus 1 (CS1). Animals were returned to their home cages immediately following the final shock in Context A.

On day 2, “O/C pair” rats were second order conditioned individually by exposing them to terpinene for 5 min continuously in Context A such that an association was formed between the odor and the context, giving odor the value of conditioned stimulus 2 (CS2). “C only” rats were simply placed in Context A for 5 min. Animals were returned to their home cages immediately following training.

On day 3 (recall day) all rats were individually placed in novel Context B and exposed to terpinene (CS2) for 5 min while recording freezing behavior.

### 2.7 First order tone conditioning

On the day proceeding habituation to Context A, experimental (tone and shock paired; T/S pair) rats were trained individually with four separate exposures to tone and shock at 5, 15, 20, and 30 min during a 30 min training session. Tone (2 kHz, 80 dB) was delivered for 1 min at each time point and co-terminated with a shock (0.5 mA for 1 s). Animals were returned to their home cages immediately following the last shock. Control rats (tone and shock unpaired; T/S unpair) were placed in Context A and given 4 shocks at 1, 3, 4, and 6 min. After 30 min, the 2 kHz tone was presented for 5 min.

The day after training (recall day) all rats were individually placed in Context A for 5 min, then exposed to the 2 kHz tone (CS) for 5 min while recording freezing behavior.

### 2.8 cFos immunohistochemical mapping

Following the final test, rats were returned to home cages undisturbed for 90 min to limit cFos expression unrelated to the behavioral experiment. Rats then received an i.p. injection of pentobarbital (200 mg/kg), and were transcardially perfused with 0.9% ice cold saline followed by 4% ice cold paraformaldehyde (PFA). Brains were carefully removed and placed in 4% PFA in glass vials for up to 1 week then transferred to PBS until sectioned.

Brains were serially sectioned coronally into 50 μm slices with a compresstome (PrecisionaryVF-210-0Z), starting from approximately −2.12 mm bregma, and collected in polyvinylpyrrolidone at 4°C for free-floating immunohistochemistry (IHC). Four to six brain sections spanning each region of the interest were selected from each animal for IHC.

Briefly, 50 μm sections were rinsed in Tris buffer and endogenous peroxidases were quenched by 1% H_2_O_2_ incubation. Sections were blocked in 10% normal goat serum with 0.1% Triton-x for 1 h prior to 4°C incubation in 1:10,000 cFos (Cell Signaling Technology, catalogue #2250S) for 2–3 days. Sections were rinsed in Tris buffer and incubated with 1:1000 biotinylated Goat anti-Rabbit IgG secondary antibody for 45 min at room temperature. The signal was amplified by an avidin-biotin peroxidase kit (ABC kit; Vector labs) for 2 h and then developed with an SG HRP substrate (SG grey; Vector labs). Sections were mounted onto slides, dried overnight under a fumehood, dehydrated by a series of ethanol and xylene, and coverslipped with Permount.

### 2.9 Image acquisition and analysis

Images were taken in the lateral amygdala (LA), anterior basolateral amygdala (BLA), posterior piriform cortex (pPC), dorsal CA1 (dCA1), dorsal CA3 (dCA3), ventral CA1 (vCA1), ventral CA3 (vCA3) and auditory cortex layers II-III (Aud_II/III_) and V (Aud_V_), with an EVOS M5000 imaging system (Thermo Fisher Scientific) and a 10X objective with consistent brightness, exposure, and gain settings (see Supplementary Figure S1 for imaging locations). Four to six images covering the rostral to caudal range of each region were analyzed and data from two hemispheres were averaged for each animal. Cells were counted automatically using ImageJ software. Images were transformed to 8-bit, converted to black and white and thresholded manually by an experimenter who remained blind to the experimental conditions. The region of interest was selected, and “despeckle” and “watershed” functions were applied to further reduce background and separate incorrectly merged cells. The cells were counted using the “analyze particles” function with size set to 15-infinity μm^2^ and circularity defined as 0.50–1.00. Cells/mm^2^ was calculated by dividing the total number of positive cells by the total area.

### 2.10 Brain tracing

For retrograde tracing experiments, Cholera Toxin subunit B (CTB) was infused bilaterally in the piriform cortex (200 nL; AP: −1.5 mm, ML: 5.6 mm bilateral, and DV: 8.7 mm from bregma) CTB-647 (0.5% w/v in phosphate buffer; Invitrogen) by a 32 g beveled 1 μL Hamilton syringe (Neuros 7001 KH) attached to a vertical infusion pump (Pump 11 Elite; Harvard Apparatus). Each infusion lasted 5 min, and remained in place for 5 min prior to syringe withdrawal. Rats were allowed 1 week for recovery before perfusion with 4% PFA. Brains were sectioned at 50 μm and cover-slipped with anti-fade fluorescence media with DAPI (abcam, catalogue # ab104139). CTB labeling in the range of DH and VH (AP 1.5 to 5.5) was examined using an EVOS M5000 system.

### 2.11 Statistics

All statistics were performed with Origin 2022b software. Freezing data and cFos expression were first tested for normal distribution using Shapiro–Wilk test. Unpaired *t*-tests were used for two group comparisons if the data were distributed normally, and non-parametric Mann–Whitney tests were used for group data that were rejected in normality tests. For group data that did not have similar variance by *F*-tests, Welch correction was applied. Data are presented as mean ± SEM.

## 3 Results

### 3.1 The VH is activated during olfactory threat memory recall while the activation of DH is contingent upon relevance of context

**Figure 1:**
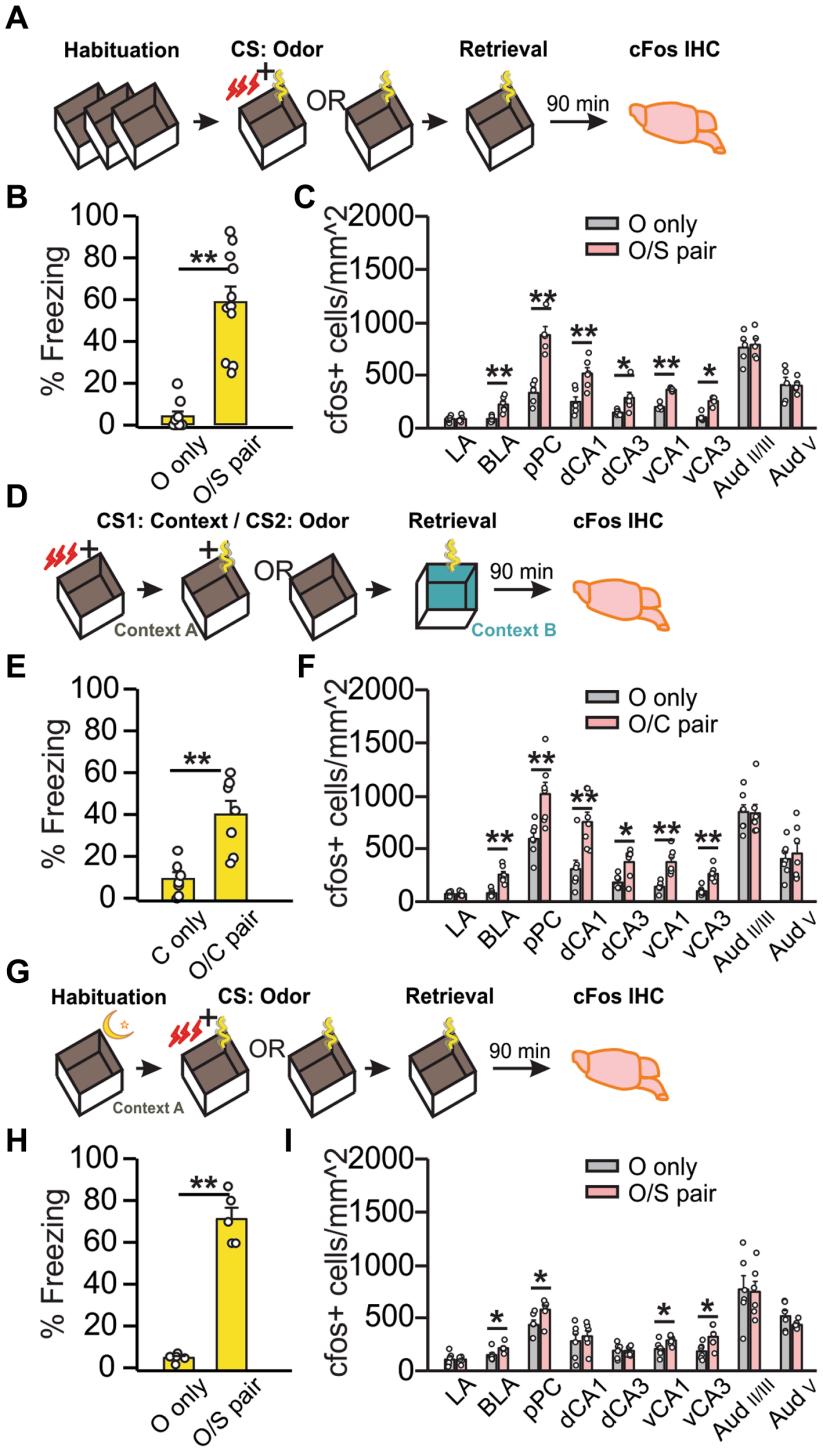
Olfactory threat memory recall activates the dorsal hippocampus only when contextual cues are relevant to the threat. **(A–C)** First order olfactory threat conditioning with standard habituation. **(A)** Schematics of the behavioral paradigm with standard habituation to Context A. **(B)** Percentage of time spent freezing during final odor exposure (time freezing/total exposure time*100%) in Context A (*N* = 10 O only, 4F/6M; *N* = 11 O/S pair, 5F/6M). **(C)** Number of cFos^+^ cells (/mm^2^) in lateral amygdala (LA), basolateral amygdala (BLA), posterior piriform cortex (pPC), dorsal CA1 (dCA1), dorsal CA3 (dCA3), ventral CA1 (vCA1), ventral CA3 (vCA3), layer II/III of the auditory cortex (Aud_II/III_), and layer V of the auditory cortex (Aud_V_) (*N* = 6 each group for LA, BLA, pPC, dCA1 and dCA3; *N* = 5 for vCA1, vCA3 and Aud). **(D–F)** Second order contextual-olfactory conditioning. **(D)** Schematics of the behavioral paradigm with no habituation to Context A during the first order contextual conditioning. **(E)** Percentage of time spent freezing during final odor exposure in Context B (*N* = 7; 3F/4M for C only; 4F/3M for O/C paired). **(F)** Number of cFos^+^ cells (/mm^2^) in LA, BLA, pPC, dCA1, dCA3, vCA1, vCA3, Aud_II/III_, and Aud_V_ (*N* = 7). **(G–I)** First order olfactory conditioning with overnight habituation. **(G)** Schematics of the behavioral paradigm with overnight habituation to Context A. **(H)** Percentage of time spent freezing during final odor exposure in Context A (*N* = 6, 3F/3M). **(I)** Number of cFos^+^ cells (/mm^2^) in the same structures as **(B,F)** (*N* = 6). *p < 0.05; **p < 0.01.

Olfactory threat conditioning was conducted following standard habituation (Figure 1A). Compared to “O only” rats, “O/S pair” rats froze for a significantly percentage of time (*U* = 0, *Z* = −3.86, *p* = 1.12E-4; Figure 1B) and expressed cFos in more cells in the anterior BLA (*t_6.33_* = −4.34, *p* = 0.004), the pPC (*t_10_* = −5.77, *p* = 4.18E-4), dCA1 (*t_10_* = −3.65, *p* = 0.0045), dCA3 (*t_5.32_* = −2.53, *p* = 0.049), vCA1 (*t_8_* = −10.57, *p* = 5.59E-6), and vCA3 (*U = 0, Z* = −2.51, *p* = 0.012; Figure 1C). The DH activation could indicate that either contextual cues were encoded to the threat memory trace or the DH participates in the recall of olfactory threat memories as part of the olfactory circuitry (Gourévitch et al., 2010). We probed these potential explanations by examining DH activation under two conditions: (1) intentional inclusion of contextual cues into the olfactory threat memory with second-order conditioning (SOC), and (2) uninterrupted context exposure through overnight habituation immediately followed by training. We used SOC to intentionally include contextual information in the odor threat memory (Figure 1D) as CS2 recall following SOC also activates CS1 representation areas in the brain (Hall, 1996). All rats were context conditioned (context CS1 + shock) with no prior habituation during phase I of the SOC. In phase II, the “O/C pair” groups were re-exposed to the context in the presence of an odor cue (CS2) to produce second-order threat conditioning, demonstrated by increased freezing behavior in response to the odor in a novel context the following day (*t_12_* = −4.19, *p* = 1.25E-3; Figure 1E). Subregional cFos expression in response to SOC threat memory recall (Figure 1F) was again enhanced in CA1 and CA3 of DH (CA1: *t_12_* = 3.56, *p* = 0.0039; CA3: *U* = 8, *Z* = −2.04, *p* = 0.041) and VH (CA1: *t_7.83_* = −4.94, *p* = 1.21E-4; CA3: *t_12_* = 5.80, *p* = 8.48E-5) in “O/C pair” rats, consistent with a role of DH in encoding of contextual cues. Finally, we minimized contextual coding by employing an overnight habituation protocol where the rats were kept in the shock chamber for 12 h followed by olfactory threat conditioning in the same context (Figure 1G). Again, “O/S pair” rats exhibited significantly more freezing than the “O only” group (*t_4.21_* = −12.06, *p* = 2.00E-4; Figure 1H). Intriguingly, when the contextual exposure was uninterrupted between habituation and conditioning, the DH was no longer activated in response to the odor threat (CA1: *t_10_* = −0.55, *p* = 0.60; CA3: *t_10_* = 0.049, *p* = 0.96), while VH activation was still observed (CA1: *t_10_* = −2.33, *p* = 0.042; CA3: *t_10_* = −2.59, *p* = 0.027; Figure 1I). Together, these experiments suggest that DH encodes contextual information, while VH is involved in olfactory threat conditioning regardless of context or DH activation. In both standard and overnight habituation protocols, pPC, BLA and VH are co-activated, consistent with the extensive mutual connections among these structures (Cenquizca and Swanson, 2007; McDonald and Mott, 2017). Projections from the VH to PC are demonstrated here by CTB retrograde tracing (Supplementary Figure S2).

### 3.2 The VH is not activated upon auditory threat memory recall

**Figure 2:**
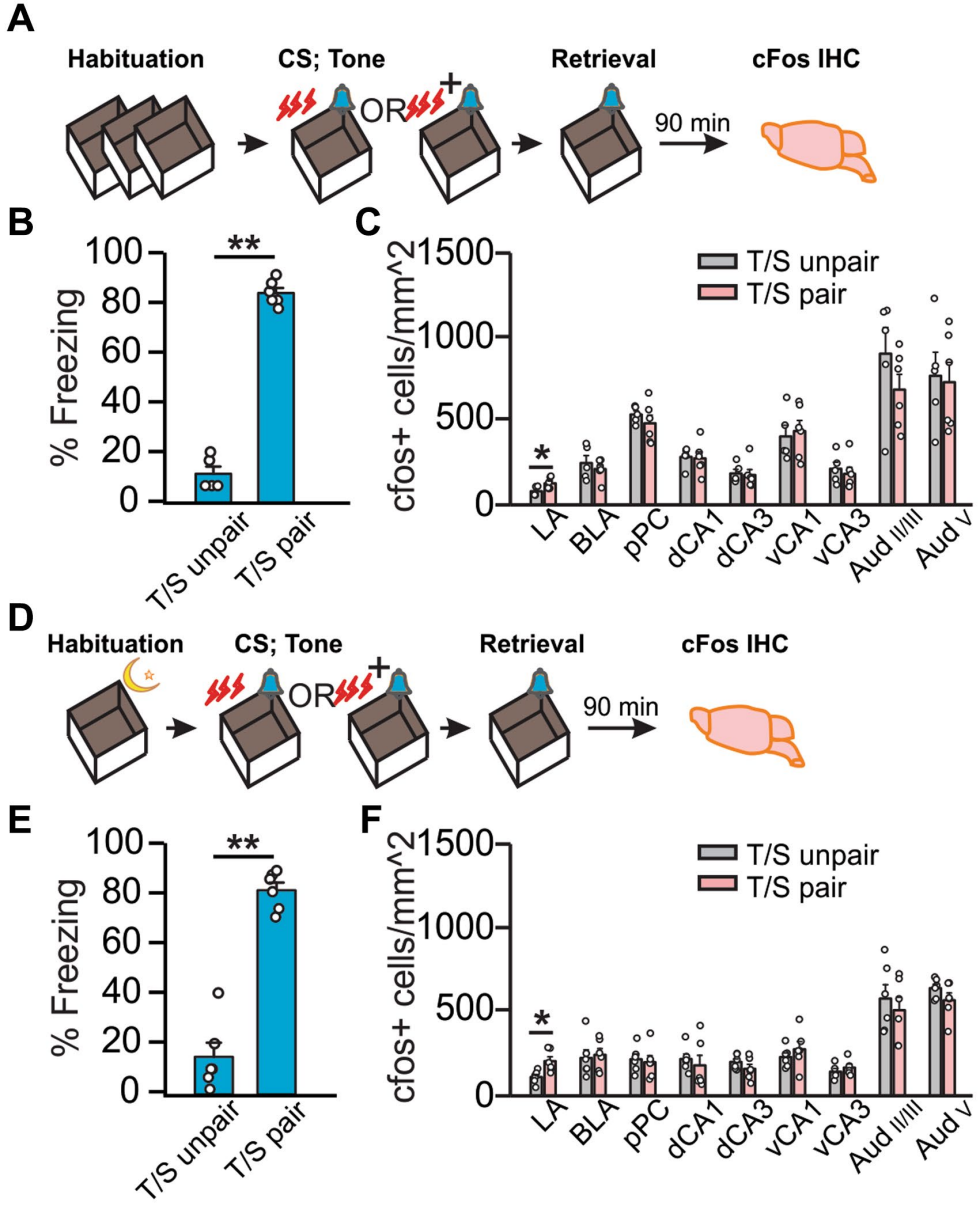
Auditory threat conditioning induced neuronal activation only in the LA. **(A–C)** Auditory threat conditioning with standard habituation. **(A)** Schematics of the behavioral paradigm with standard habituation to Context A. **(B)** Percentage of time spent freezing during final tone exposure (time freezing/total exposure time*100%) in Context A (*N* = 5 T/S unpaired; *N* = 6 T/S paired). **(C)** Number of cFos^+^ cells (/mm^2^) in LA, BLA, pPC, dCA1, dCA3, vCA1, vCA3, Aud_II/III_, and Aud_V_ (*N* = 5 T/S unpaired, 3F/2M; *N* = 6 T/S paired, 3F/3M). D-F. Auditory conditioning with overnight habituation. **(D)** Schematics of the behavioral paradigm with overnight habituation to the context. **(E)** Percentage of time spent freezing during final odor exposure in Context A (*N* = 6, 4F/2M for T/S unpaired, 3F/3M for T/S paired). **(F)** Number of cFos^+^ cells (/mm^2^) in LA, BLA, pPC, dCA1, dCA3, vCA1, vCA3, Aud_II/III_, and Aud_V_ (*N* = 6). *p < 0.05.

We next tested if VH activation is involved in auditory threat conditioning. To mitigate cFos expression related to context, we used a design with equivalent exposures to shock, context, and sensory cues between “T/S paired” and “T/S unpaired” groups. Following Hsd (Figure 2A), “T/S paired” rats spent a higher percentage of time freezing than the “T/S unpaired” rats (*U = 0, Z*_=_ − 2.67, *p* = 0.007; Figure 2B) in response to the conditioned tone. Intriguingly, only cFos expression in the LA was significantly higher in the “T/S paired” group (*t_9_* = −2.48, *p* = 3.51E-2; Figure 2C), consistent with the role of the LA in auditory threat conditioning (Quirk et al., 1995; Nader et al., 2001). Because both groups of rats were shocked in the context in which they were tested, the opportunity for contextual conditioning was equal across groups, and cFos activation between groups is likely specific to the auditory cue. Even though the “T/S unpaired” rats showed a significantly lower percentage of freezing than the “T/S paired” rats, the freezing level was higher than the odor only rats with the same standard habituation method (Supplementary Figure S3), suggesting weak context conditioning occurred with these rats. Overnight habituation with auditory threat conditioning (Figure 2D) yielded similar results. “T/S paired” group spent more time freezing than the control group (*t_10_* = −10.32, *p* = 1.19E-6; Figure 2E) and increased cFos was observed in the LA (*t_10_* = −3.07, *p* = 1.18E-2; Figure 2F). Conversely, cFos expression did not differ between groups in all other regions in either of the habituation conditions (Figures 2C,F), suggesting that the hippocampus is not critically involved in the recall of auditory threat memory.

## 4 Discussion

Auditory (Goosens and Maren, 2001), visual (Bergstrom and Johnson, 2014), and olfactory (Cousens and Otto, 1998) threat memory reactivation recruits distinct yet overlapping brain areas. The use of shock, a strong aversive unconditioned stimulus (Azrin et al., 1967), requires that an animal be habituated to every aspect of an experimental setup except the cue and unconditioned stimulus prior to training (Curzon et al., 2009) to ensure the specificity of the threat memory to the conditioned sensory stimulus and not the experimental context. A cue is comprised of sensory features, while context includes the sensory features of the environment in addition to temporal, spatial, and internal factors such as an animal’s cognitive, hormonal, and motivational state (see Maren et al., 2013 for review). For experimental purposes, the concept of context is often simplified to include only external elements of the physical environment. Context itself can be conditioned to elicit a defensive response, a process routinely employed as a standard threat conditioning paradigm (Phillips and LeDoux, 1992; Anagnostaras et al., 1999; Goosens and Maren, 2001; Curzon et al., 2009; Maren et al., 2013; Orsini et al., 2013).

Over the past several decades, the hippocampus has emerged as a critical structure for learning contextual information. Lesions in this area tend to inhibit the expression of freezing behavior in response to a context previously paired with shock, especially when performed 24 h after conditioning, but not necessarily if performed prior to conditioning (Maren et al., 1997;Frankland et al., 1998; Wiltgen et al., 2006). Consequently, it has been proposed that individual elements of a context are represented elsewhere in the brain and that these representations are sufficient for learning in the absence of a functioning hippocampus (see Maren et al., 2013 for review). Instead, the hippocampus serves to link these individual elements of the context to form a configural representation rather than to encode context-US associations directly, and it is this configural representation that is activated upon contextual threat memory recall (Maren et al., 1997; Fanselow, 2000; Maren, 2001). Evolutionarily speaking, encoding a configural representation of a specific context in which an aversive experience occurred would be important for survival, as this configural representation or gestalt of a context allows an animal to frame a given threat memory using a variety of aspects from previous experiences to inform future adaptive behavior (Maren et al., 2013). When the hippocampus is lesioned after conditioning, the previously encoded configural representation is inaccessible, and no threat response is observed (Frankland et al., 1998; Wiltgen et al., 2006). Further, observations that immediate shock prevents contextual threat memory encoding (Fanselow, 1990) support this theory, as there is insufficient time to encode a configural representation of the context prior to shock (Fanselow, 1986; Frank et al., 2004).

Our results and those of others imply that context encoding is likely universal in cued threat conditioning. A PET imaging study investigated the response to a recent olfactory threat memory found regional activation patterns in agreement with those in this study under standard habituation. Importantly, no habituation to the context prior to training occurred, instead the authors included additional contextual elements during the testing phase to create a “novel” context intended to exclude any responses to the training context. They explained that the DH activity upon recall was likely due to recognition of parts of the conditioning context that had not been altered (Mouly et al., 2022). These findings illustrate how each encounter of a particular context may be similar yet distinct, as context is a unique combination of the external environment and internal state of the animal (Maren et al., 2013). The results described here suggest that standard habituation protocols for cued conditioning may be insufficient if contextual conditioning is not desired. We have described two ways to mitigate contextual conditioning: overnight habituation prior to training such that the same contextual information prior to training is preserved during the threat conditioning, or use of a control group with equal opportunity for contextual conditioning. We have demonstrated how these manipulations exclude the DH from the memory trace, suggesting that DH activation could serve as an indicator of contextual encoding in cued threat learning.

The longstanding notion of functional homogeneity along the dorsal-ventral hippocampal axis has been dismissed by numerous observations that the ventral, but not dorsal, hippocampus plays a critical role in threat learning (Bast et al., 2001; Kjelstrup et al., 2002; Maren and Holt, 2004) and anxiety-like behavior (Adhikari et al., 2010; Deji et al., 2022). This is likely through its extensive reciprocal connections with the amygdala (McDonald and Mott, 2017; Deji et al., 2022), where the encoding of both cue- and context-US associations takes place (LeDoux, 2000; Davis and Whalen, 2001; Maren and Quirk, 2004; Fanselow and Poulos, 2005). Dorsal and ventral subregions are extensively connected by intrahippocampal circuits, but direct connections to the amygdala, originate solely from ventral CA1 and subiculum (see McDonald and Mott, 2017 for review). As such, any direct projections from DH to the amygdala must travel through the VH, potentially obfuscating the precise roles of DH and VH in threat conditioning from early studies that tended to rely on anatomical lesions.

Lesions of the VH impair the discrimination of olfactory, but not visual or spatial (Hunsaker et al., 2008) cues, consistent with our finding that the VH is active upon olfactory, but not auditory, threat memory recall. Numerous observations demonstrate the dense interconnectivity of the ventral CA1 with primary olfactory areas (Cenquizca and Swanson, 2007; Padmanabhan et al., 2018), which is confirmed by CTB retrograde tracing in this study (Supplementary Figure S2). To our knowledge, projections from the ventral CA1 to the primary auditory cortex are sparse (Cenquizca and Swanson, 2007). Differential activation of the VH in threat conditioning with olfactory vs. auditory cues may be explained by the difference in neural processing of these two distinct sensory modalities, namely that information about sound is processed by the thalamus prior to reaching the sensory cortex while information about odor can reach the cortex directly without thalamic relay (Kandel et al., 2014). Rats with lesions to the auditory cortex can still form threat memories to simple tones, but not to complex sounds (Peter et al., 2012), and temporary inactivation of the VH but not DH impairs the acquisition but not recall of threat memories to pure tones (Maren and Holt, 2004). Both of these observations are in agreement with our cFos data illustrating a lack of activation in the auditory cortex and VH between paired and unpaired groups upon recall of a threat memory to pure tone (Figures 2C,F). It is reasonable to hypothesize then that the VH is differentially involved in the encoding and subsequent recall of threat memories with different sensory modalities. The increased activation in the LA that we observed in response to auditory threat memory recall is consistent with the known role of the LA in the formation of CS-US associations during auditory threat conditioning (Quirk et al., 1995; An et al., 2012; Bergstrom and Johnson, 2014), but lack of activity in VH and auditory cortex suggests that the information may be reaching the lateral amygdala by an alternate, possibly thalamic, route. Indeed, several observations suggest that the medial geniculate nucleus modulates the encoding of auditory threat learning with pure tones in the amygdala (Cruikshank et al., 1992; Weinberger, 2011; Ferrara et al., 2017).

In summary, our data suggest that the DH encodes contextual information in cued threat conditioning but does not participate directly in either olfactory or auditory threat memory recall. Conversely, the VH is involved in olfactory, but not auditory, threat memory. We propose that activation of the DH may serve as an indication that contextual information is associated with a particular cue-dependent threat memory, and offer two strategies to mitigate contextual encoding. Olfactory pathway-specific silencing or activation of the VH during threat conditioning or recall may further illuminate the role of the VH in the encoding and expression of olfactory threat memory.

## Data availability statement

The original contributions presented in the study are included in the article/Supplementary material, further inquiries can be directed to the corresponding authors.

## Ethics statement

The animal study was approved by the Memorial University Institutional Animal Care Committee. The study was conducted in accordance with the local legislation and institutional requirements.

## Author contributions

TS: Data curation, Formal analysis, Writing – review & editing. SC: Data curation, Formal analysis, Writing – original draft, Writing – review & editing. QY: Conceptualization, Funding acquisition, Project administration, Supervision, Visualization, Writing – review & editing.
