# Supplementary material for: The ventral hippocampus is activated in olfactory but not auditory threat memory
Source: Front Neural Circuits. 2024 Feb 27;18:1371130. doi: 10.3389/fncir.2024.1371130 (PMC10927826; doi:10.3389/fncir.2024.1371130)
Supplement: Supplementary file 1 [file Data_Sheet_1.PDF]

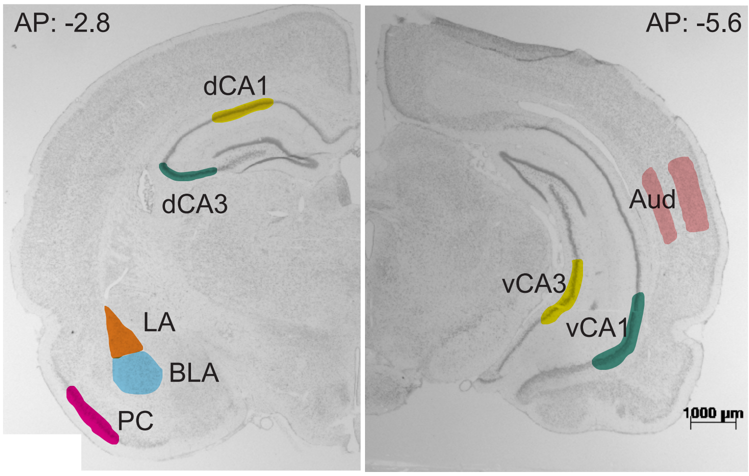

Supplementary Figure 1. Imaging areas for cFos analysis.

PC: piriform cortex. LA: lateral amygdala. BLA: basolateral amygdala. dCA1: dorsal CA1. dCA3: dorsal CA3. vCA1, ventral CA1. vCA3, ventral CA3. Aud: Auditory cortex.

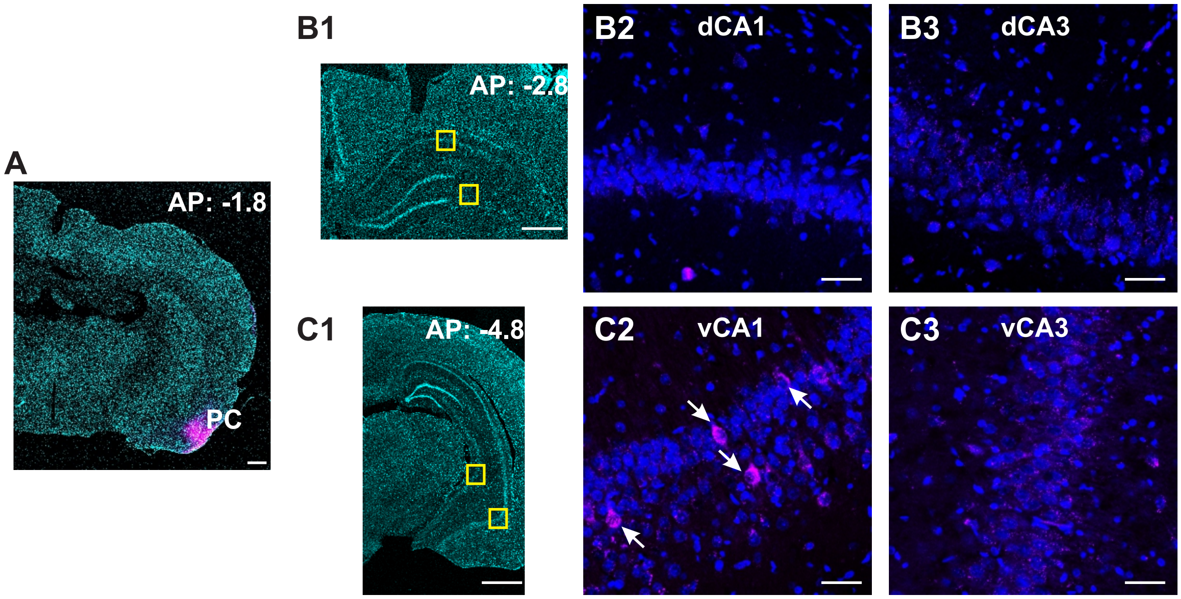

Supplementary Figure 2. The ventral hippocampus projects to the piriform cortex.

A. CTB in the piriform cortex (PC). Scale bar: 1000 µm. **B1-B3.** No labeled cells were observed in the dorsal hippocampus (dCA1 and dCA3). B2-B3 are zoom in images of the regions in the yellow squares in B1. **C1-C3.** Labeled cells were observed in the ventral CA1 (vCA1). We examined total 23 brains with PC CTB infusions. Three of these brains that are off targeting and two with poor tissue quality were excluded from analysis. Out of the 18 brains, we observed 57.39±9.08 cells in a single section showing the maximum CTB labeling (~AP-4.8 to -5.0). CTB labeled cells are mainly located in the ventral 1/3 of the VH. C2-C3 are zoom in images of the regions in the yellow squares in C1. White arrows indicated labeled cells (magenta). Scale bars in B1 and C1: 500 µm; in B2, B3, C2, C3: 50 µm.

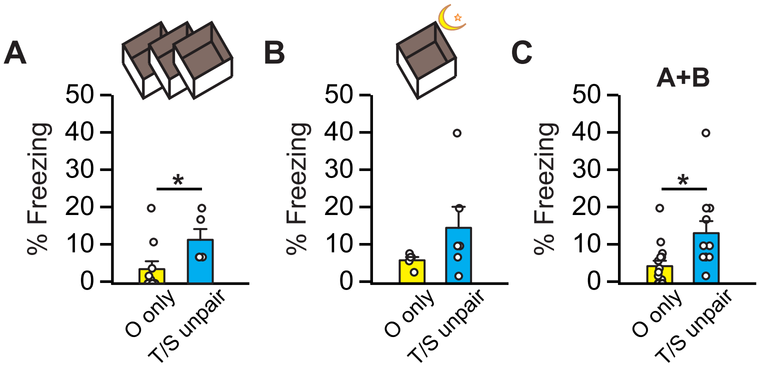

Supplementary Figure 3. Weak context conditioning in T/S unpaired rats.

A. Comparison of percent freezing in odor only rats and T/S unpaired rats underwent standard habituation. **B.** Comparison of percent freezing in odor only rats and T/S unpaired rats underwent overnight habituation. **C.** Comparison of percent freezing in odor only rats and T/S unpaired rats in both habituations. *$p < 0.05$
